# Health and economic effects of air pollution in the USA and India: A comparative study of two nations' breath

**DOI:** 10.3934/publichealth.2025044

**Published:** 2025-09-05

**Authors:** Arvind Goswami, Harmanpreet Singh Kapoor, Ankita Sharma, Vivek Jadhav, Rajesh Kumar Jangir, Vijay Kumar Chattu

**Affiliations:** 1 Department of Economic Studies, Central University of Punjab, Ghudda (Bathinda), 151401, India; 2 Department of Mathematics and Statistics, Central University of Punjab, Ghudda (Bathinda), 151401, India; 3 Independent Consultant, PhD Health Economics, Manipal University Jaipur, Rajasthan, India; 4 Department of Economic Environment and Policy, Institute of Management Technology, Ghaziabad-201001, India; 5 Center for Economic Studies and Planning, School of Social Sciences, Jawaharlal Nehru University, New Delhi, India; 6 Department of Public Health, Health Administration & Information and Health Sciences, College of Health Sciences, Tennessee State University, Nashville, TN 37203, USA; 7 Department of Epidemiology and Biostatistics, Semey Medical University, Semey, 071400, Kazakhstan; 8 Department of Community Medicine, Faculty of Medicine, Datta Meghe Institute of Medical Sciences (DMIMS), Wardha 442107, India

**Keywords:** air pollution, mortality, cancers, heart diseases, health economics, economic loss, USA, India

## Abstract

**Background:**

Air pollution is a leading cause of premature deaths in developing countries compared to developed countries. We aimed to analyze and compare the economic loss due to premature deaths caused by air pollution in the USA and India.

**Methods:**

Data on household and ambient air pollution, mortality, population, and GDP were collected from the WHO Global Health Observatory, the 2019 Global Burden of Disease Study, and World Development Indicators. The economic loss of premature deaths caused by air pollution were assessed for 2019 in India and the USA by calculating the adjusted labor output per worker, factoring in the likelihood of a person being employed. However, reported mortality cases of air pollution can be less than the actual cases, so the actual loss can be greater than that calculated in this study.

**Results:**

The findings showed that in 2019, the total economic loss due to premature deaths caused by air pollution was $34.85 billion and $24.76 billion in India and the USA, respectively. In 2019, India and the USA lost around 1.67 million and 100,000 lives because of air pollution, respectively. However, the per capita loss amounted to $20,868 for India and $247,600 for the USA, highlighting the stark disparity in the per capita income. Despite significant socioeconomic variations, ambient air pollution is the leading cause of total premature deaths from air pollution, accounting for 58% and 80% in India and the USA, respectively.

**Conclusions:**

Air pollution is rising in India and decreasing in the USA. The United States has implemented stringent laws and regulations, such as the Clean Air Act, to control air pollution, and India should benefit from this example. Moreover, monitoring the ground-level situation is important to reduce air pollution and associated fatalities.

## 1. Introduction

Air pollution is ranked the fourth leading risk factor for human health globally, followed by high blood pressure, dietary hazards, and smoking [1],[2]. It is a major cause of premature death and disease and is the largest environmental health threat in the twenty-first century [3],[4]. Air pollution is also associated with three principal causes of death worldwide: Stroke (26%), ischemic heart disease (20.2%), and primary cancer of the trachea, bronchus, and lung (19%). Air pollution is considered a significant threat to humans; for instance, globally, only one of ten people living in areas where air quality is as per recommended air quality levels [3]. Responsible for seven million fatalities annually, representing one in every eight deaths globally [3], air pollution surpasses all other major preventable causes of mortality, including tobacco use, alcohol use, traffic accidents, and transmissible diseases like HIV/AIDS, malaria, and tuberculosis. Tiny particulate matter, especially PM_2.5_ pollution, in the ambient air, can lead to adverse health effects, including respiratory irritation, pulmonary dysfunction, and cardiovascular disease, potentially resulting in death or morbidity. Moreover, PM_2.5_ pollution imposes higher economic costs, such as reduced worker productivity and increased healthcare expenditures [1],[3],[5].

The economic cost of air pollution has been borne more by developing countries than by developed countries. For instance, in India, the total number of deaths due to air pollution in 2019 was approximately 1.67 million [6] compared to the United States of America (USA), where it was around 100,000–200,000 [7]. Air pollution-related deaths in India are higher than in the USA because of several factors deeply embedded in the nation's socio-economic structure [8]. India's swift industrialization and urbanization have resulted in an abundance of automobiles, factories, and construction endeavors, all of which have markedly increased air pollution levels [9]. The widespread use of biomass burning for cooking and heating has also worsened the issue, particularly in rural areas [10]. The problem is exacerbated by insufficient enforcement and regulatory procedures, which permit industries to function without strict emissions limits. Additionally, the high population density and inadequate infrastructure for waste management and sanitation create fertile ground for pollutants to accumulate [11]. Even though the USA has its problems with air pollution, such as emissions from cars and industrial operations, the effects are lessened by stronger laws and enforcement systems.

The Fourth National Climate Assessment projected that the USA would see 500 fewer premature deaths annually due to air pollution by 2090. However, the situation is different in India, where the burden of PM_2.5_ is anticipated to increase due to climate change. While actions to mitigate climate change can mitigate health impacts by addressing the climate penalty and its effects, estimations of these co-benefits remain uncertain [12],[13]. In developing countries like India, vehicle emissions frequently contribute to air pollution (40%–80%) [14]. The leading causes of this pollution are ambient particulate matter (PM_2.5_ and PM_10_) [14]–[16], residential air pollution, and, to a lesser extent, tropospheric ozone [4],[17]. In India, coal combustion for thermal power generation, industrial emissions, construction activities, brick kilns, vehicles, road dust, biomass burning for residential and commercial purposes, waste incineration, agricultural residue burning, and diesel generators are the significant sources of ambient particulate matter pollution [4],[15],[17].

Few researchers highlighted that due to poor air quality, the thin particles accumulate in human lung tissue and disrupt normal function, resulting in lung disease or non-specific functional alterations, such as acute respiratory infection (ARI), asthma, bronchitis, or a loss in lung capacity [14],[18],[19]. Moreover, India's annual average exposure to ambient particulate matter PM _2.5_ is among the highest in the world, with 77% of the population having mean levels over 40 go/m³, exceeding the National Ambient Air Quality Standards set by the World Health Organization (WHO) in 2017 [17]. Between 1990 and 2019, residential air pollution in India declined drastically, whereas ambient particle matter and ozone pollution rose. In 2019, less developed regions in north and northeastern India had a more significant burden of air pollution, whereas northern states had a higher burden of ambient particulate matter pollution. The economic cost of early deaths and illness due to air pollution was substantial, accounting for 1.36% of India's Gross Domestic Product (GDP) in 2019 [20]. According to National Health Accounts data, India's overall healthcare expenditure in 2019 was $103.7 billion. Furthermore, in 2019, air pollution caused 11.5% of India's disease burden (measured as disability-adjusted life years or DALYs), which resulted in an estimated healthcare expenditure of $11.9 billion [4].

Since 1970, the Clean Air Act has significantly reduced emissions of designated and ambient pollutants, with a 31% reduction in aggregate criterion emissions between 1970 and 1997, with variations ranging from a 98% drop in lead emissions to a slight rise in NO_2_ emissions among the US population [21]. A study conducted by the researchers in [22] in the USA found that 41%–53% of premature deaths caused by the US air quality-related emissions occur outside of states. Electric power generation has the most significant influence, whereas commercial/residential emissions have the least. Since 2005, reductions have increased premature mortality in the commercial and residential sectors. Nitrogen oxides (NO_x_) and sulfur dioxide (SO_2_) emissions were the leading causes of early mortality [22]. In 2018, greenhouse gas and hazardous air pollutant emissions caused the loss of 388,000 DALYs. There was significant heterogeneity in state-level greenhouse gas emissions per capita, which were not strongly associated with healthcare quality in the US [22].

According to the Environmental Protection Agency (EPA), in 2023, 140 million Americans, or 40% of the total population, resided in areas with poor air quality [23]. People reacted to air pollution by avoiding outside activities and spending more money on face masks, air purifiers, and health insurance. This resulted in an increase in expenditure on these items [24]. A review highlighted that the European Air Quality Limit Values (AQLVs) and National Ambient Air Quality Standards (NAAQS) are effective models for planning and reducing emissions. European rules identify risk exposure zones, while the US sets universal air quality requirements based on severity levels. Both models have contributed to significant reductions in emissions and associated health and environmental consequences [25].

This study examines the impact of air pollution on public health and economic prosperity in the United States and India. The objective of this study is to compare and contrast India, a developing economy, with the USA, a developed economy. Comparing the economic and health costs of mortality from air pollution in developed and developing economies is essential to determine the extent of the problem and setting priorities for successful intervention measures. Researchers have examined the economic losses in India and Africa [26] and one or a small number of economies with similar socio-economic circumstances. However, no research comparing the economic and health effects of air pollution between developed and developing economies has been done. Furthermore, other economy can benefit from the lessons that can be drawn by contrasting the USA and India. Additionally, lessons learned from comparing India and the USA can be applied to other countries. This comparison can help inform international efforts to reduce air pollution and mitigate its impacts on human health and the environment. This will also provide valuable insights into the disparities in healthcare systems, environmental regulations, and socio-economic factors contributing to the varying economic burdens. Additionally, understanding the economic burden in developed and developing countries would help policymakers allocate resources appropriately and implement targeted interventions to address the specific challenges faced by each region.

## 2. Research methodology and data

In this study, the methodology for estimating economic losses associated with air pollution-related mortality builds upon the approach utilized by Fisher et al. (2021) [26]. Their model estimates lost labor income per worker, adjusted for age-specific employment probabilities, effectively capturing both market and non-market production losses. This makes it particularly useful for evaluating economic impacts across varying socio-economic settings [27],[28]. The WHO Global Health Observatory database was used to collect data on the concentration and trend of household and ambient air pollution. The 2019 Global Burden of Disease Study (GBD) was used to collect information about mortality related to air pollution [29]. Data on population and GDP were collected from World Development Indicators [30].

The model's systematic approach to calculating the present discounted value of future economic losses due to premature mortality supports detailed cross-country comparisons, even when pollutant-specific data are constrained. Additionally, the model's adaptability to differing data conditions enhances its applicability in contexts like India, where reporting gaps and limited pollutant monitoring might otherwise impede comprehensive economic assessments.

### 2.1. Determination of monetary loss to the economy due to air pollution mortality

The estimation of the present discounted value of the loss in GDP associated with mortality due to air pollution is provided. The data has been collected for the year 2019. The loss in GDP due to the loss of labor force for India is evaluated by taking labor's contribution to the GDP (*αY_i_*) divided by the total workforce (*L_i_*) with the assumption that all the workers of all age groups are equally efficient or equally contributing to the economy. As all the persons of the same age group *j* do not contribute to GDP, it is essential to derive the expected value per worker for a person age *j* (*W_ij_2019*) is equal to (*αY_i_/L_i_*) times the ratio of the number of workers of age j, L_ij_, to the population of age j, N_ij_. The mathematical expression is explained below:

$$W_{ij2019}=\left(\frac{\alpha Y_{i}}{L_{i}}\right)*\left(\frac{L_{ij}}{N_{ij}}\right)$$ (1)

where *W_ij_*_2019_ represents the expected value of GDP per worker with age *j*.

It is assumed that the share of labor to the GDP (α) is constant over time and $\left(\frac{{\text{L}}_{\text{ij}}}{{\text{N}}_{\text{ij}}}\right)$ remains fixed over time. The value of α as per Our World in Data is 57.8 and 60.4 percent in India and the USA, respectively. Further, eq 1 is modified to allow for households and calculate the market and non-market output loss in 2019. The modified expression is discussed as:

$$W\prime_{ij2019}=\left(\frac{\alpha Y_{i}}{Li}\right)*\left(\frac{L_{ij}}{N_{ij}}\right)+\lambda_{j}\left(\frac{\alpha Y_{i}}{L_{i}}\right)*\left[1-\left(\frac{L_{ij}}{N_{ij}}\right)\right]$$ (2)

where *λ*_*j*_ represents the proportion of output associated with non-market production for a person of age *j*, which is 30% in the case of India and 25% in the USA.

The contribution to GDP will be lost for all future years of their working life when a person of age *j* dies in that year. The country's GDP grows at some rate, so to compute the loss amount of GDP in monetary terms, a constant growth rate *g_i_* has been assumed for the country *i*. Assuming labor's share of GDP and the proportion of the population of working age *L_ij_*/*N_ij_* constant, then the loss of GDP at age t of an individual having age *j* can be derived as:

$$\left(\frac{\alpha Y_{i}}{L_{i}}\right)*\left(\frac{L_{it}}{N_{it}}\right)*{\left(1+g_{i}\right)}^{t-j}$$ (3)

The probability of survival of an individual of age *j* in country *i* survives to age *t* is denoted by *π_ij_*_,*t*_. The probability of death of an individual of age *j* is given in the interval of 5 years (0–4, 5–9..., 75–79) from life tables. Each class interval's probability is deducted from 1 to get the survival probability of that interval. The following concept of probability has been used to derive the probability of survival for a person of age *j* to age t:

$$\pi_{ij,t}=\pi_{i,j,j+4}\ldots .\pi_{t-4,t}$$ (4)

where the *π_i_*_,*j*,*j*+4_ denotes the probability of survival of an individual age *j* in the age interval *j* to *j* + 4. Similarly, *π_t_*_−4,*t*_ is the survival probability of an individual in the age interval *t* − 4 and *t*.

The value of GDP is discounted for future loss at the annual rate *r_i_*.

Using all the information given, the present discounted value of lost market and non-market output of an individual age *j* in country *i* who dies in the year 2019 is derived as:

$$PV_{ij}=\overset{80}{\underset{t=j}{\sum }}\pi_{ij,t}\left[\left(\frac{\alpha Y_{i}}{Li}\right)*\left(\frac{L_{ij}}{N_{ij}}\right)+\lambda_{j}\left(\frac{\alpha Y_{i}}{L_{i}}\right)*\left[1-\left(\frac{L_{ij}}{N_{ij}}\right)\right]\right]{\left(\frac{1+g_{i}}{1+r_{i}}\right)}^{t-j}$$ (5)

Equation 3 is calculated for *j* = 0,...,80.

The product of *PV_ij_* and *D_ij_* denotes the total output loss due to air pollution mortality, where *D_ij_* denotes the number of deaths due to air pollution in 2019 of persons of age *j* in country *i*, summed over all *j*. For each category of air pollution, *D_ij_* is computed separately.

## 3. Results

Ambient air pollution is the leading air pollution in the USA and India, but it is declining in the USA, whereas in India, it is increasing. In 2019, the average population-weighted mean PM_2.5_ concentration, used to measure ambient particulate matter exposure, was 91.7 µg/m³ in India and 7.66 µg/m³ in the USA. As per the WHO guidelines, the annual concentration of PM_2.5_ should be less than 5 µg/m³ [31]. The higher concentration level can lead to diseases. In the case of India, the situation is alarming, with an average PM_2.5_ concentration exceeding WHO guidelines by over 18 times. Moreover, the USA's situation is much better than India's, though its concentration level is also higher than the prescribed limit by the WHO. The impact of this can be seen through the results of the study, where both the economic and human losses are higher in India. India's total economic loss due to premature deaths attributed to air pollution in 2019 was $34.85 billion, which was 1.29% of total GDP. In the case of the USA, the total economic loss was $24.76 billion, which was 0.12% of the economy. The outcomes show that the economic loss caused by air pollution is higher in India than in the USA, which indicates that developing nations bear more burden of air pollution than developed nations. As per the data from India, deaths due to air pollution per 100,000 population are approximately 164, compared to the USA, where it is only 10.55. However, the USA's per capita loss is three times India's per capita loss.

**Figure 1.**
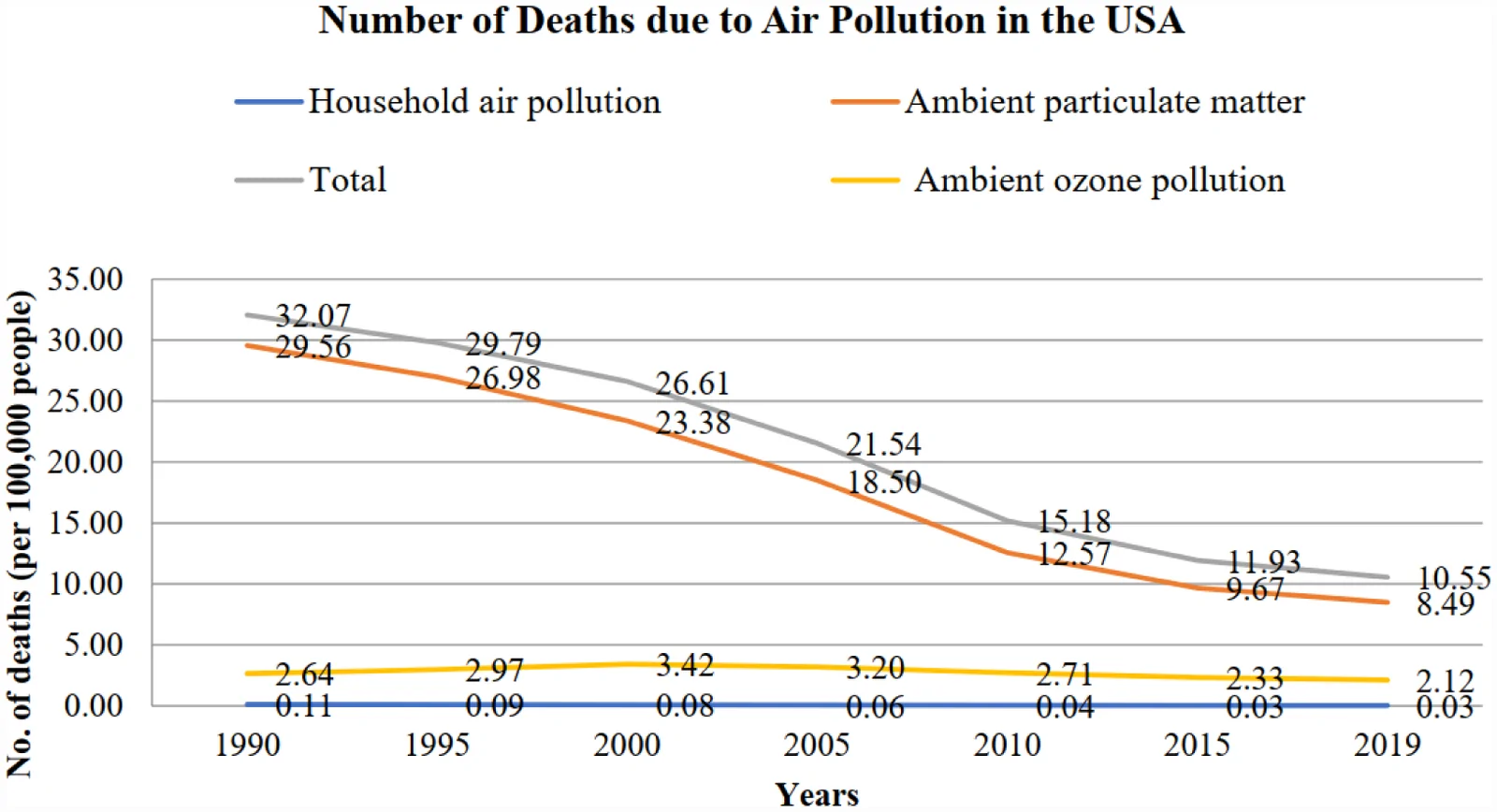
Number of deaths due to different forms of air pollution in the USA (source: Prepared by authors).

**Figure 2.**
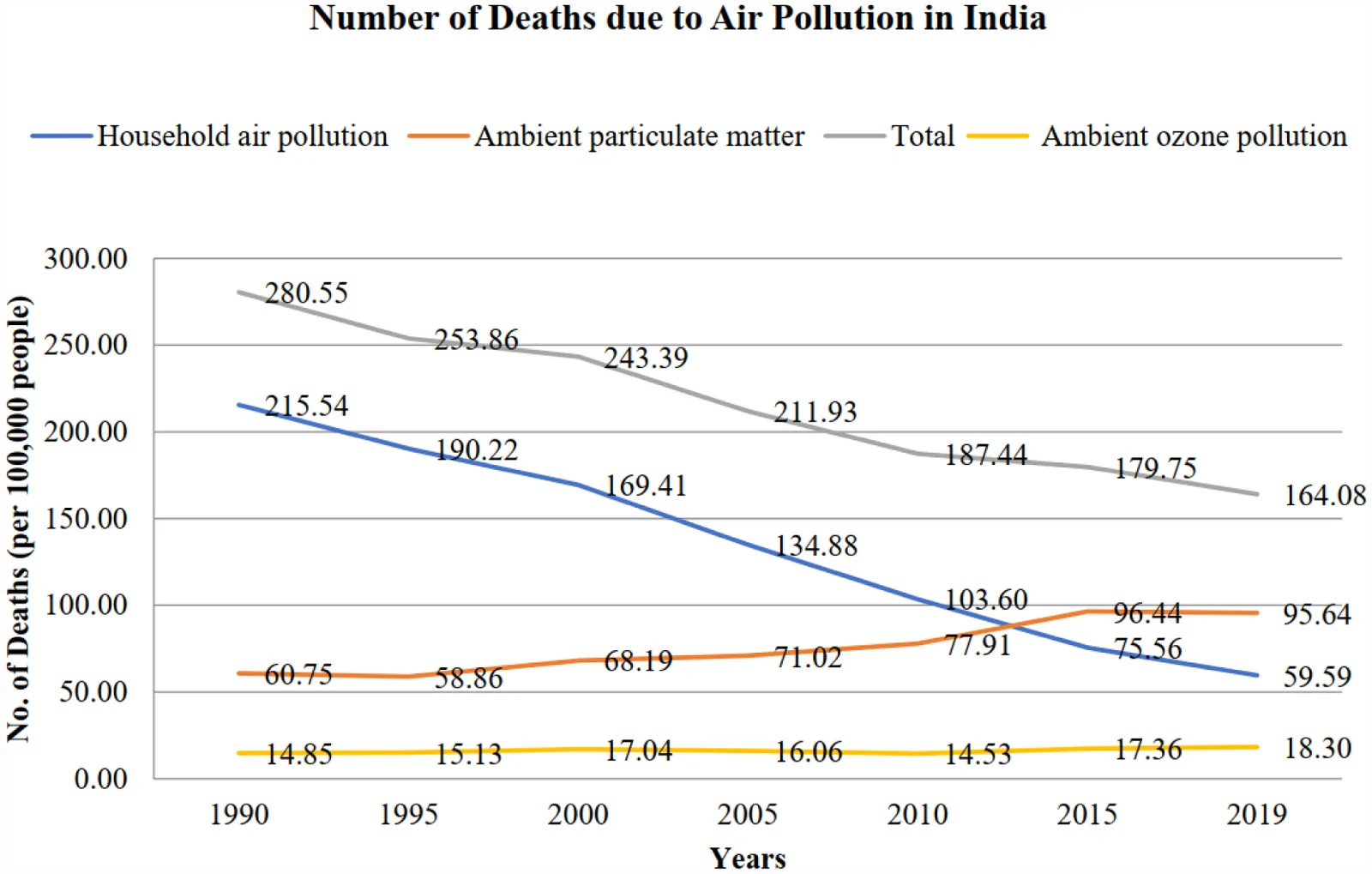
Number of deaths due to different forms of air pollution in India (source: Prepared by authors).

Nevertheless, the large difference between the number of deaths shows the air pollution level in India, with indoor air pollution having long been the predominant concern until 2012, being overtaken by ambient air pollution. By 2019, the toll of air pollution-related fatalities had reached a staggering 1.7 million in India, with 58% attributed to outdoor particulate matter, 37% to indoor air pollution, and 11% to outdoor ozone pollution. In contrast, the United States has mostly contended with ambient air pollution for decades. However, the American scenario presents a markedly improved picture compared to India. In 2019, the total number of deaths attributable to air pollution in the USA stood at approximately 100,000, less than 10% of total deaths in India. Among these, 80% were linked to outdoor particulate matter, 19.97% to outdoor ozone pollution, and a mere 0.03% to indoor air pollution, which are depicted in Figures 1 and 2.

**Figure 3.**
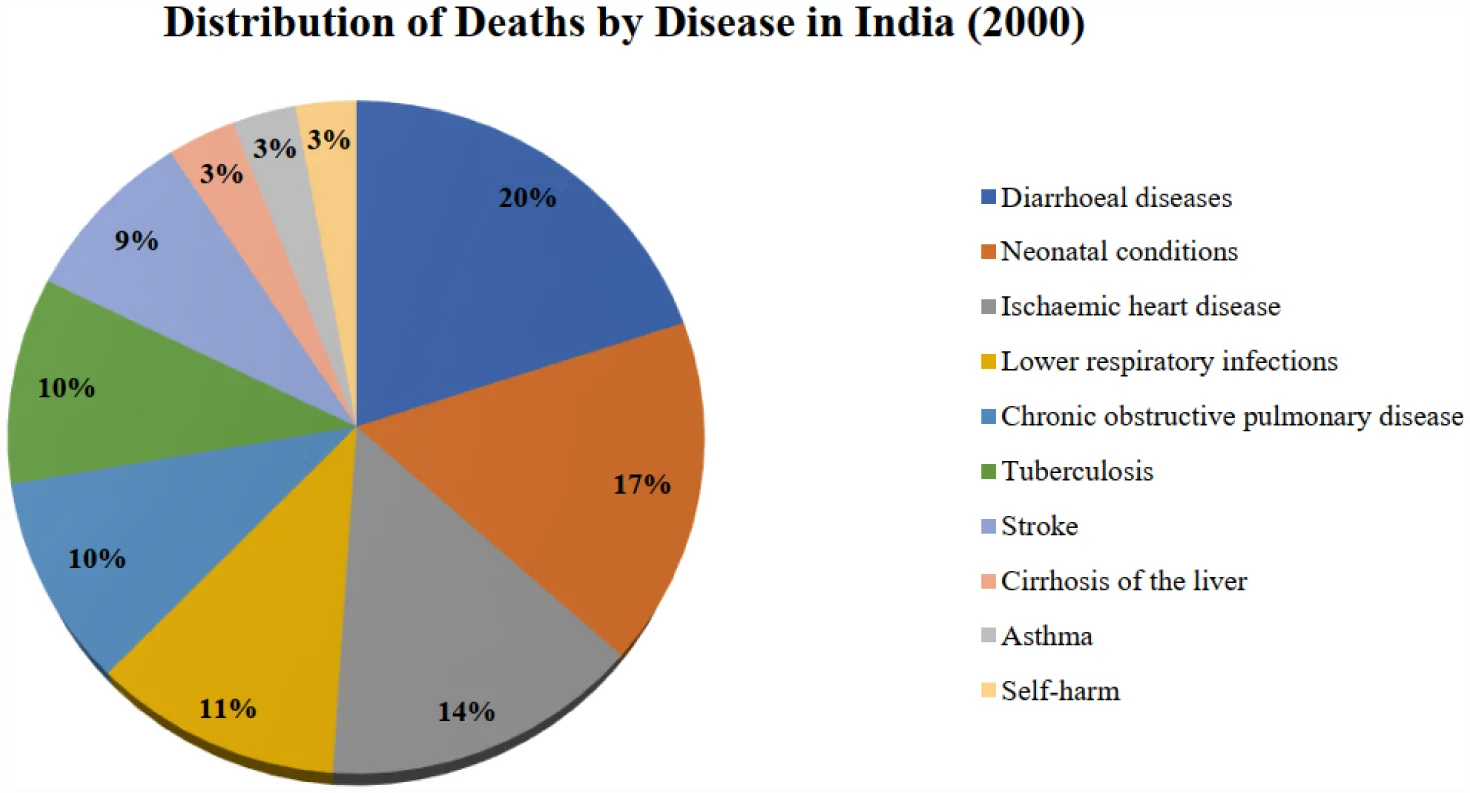
Percentage distribution of deaths by disease in India, 2000 (source: Prepared by authors).

**Figure 4.**
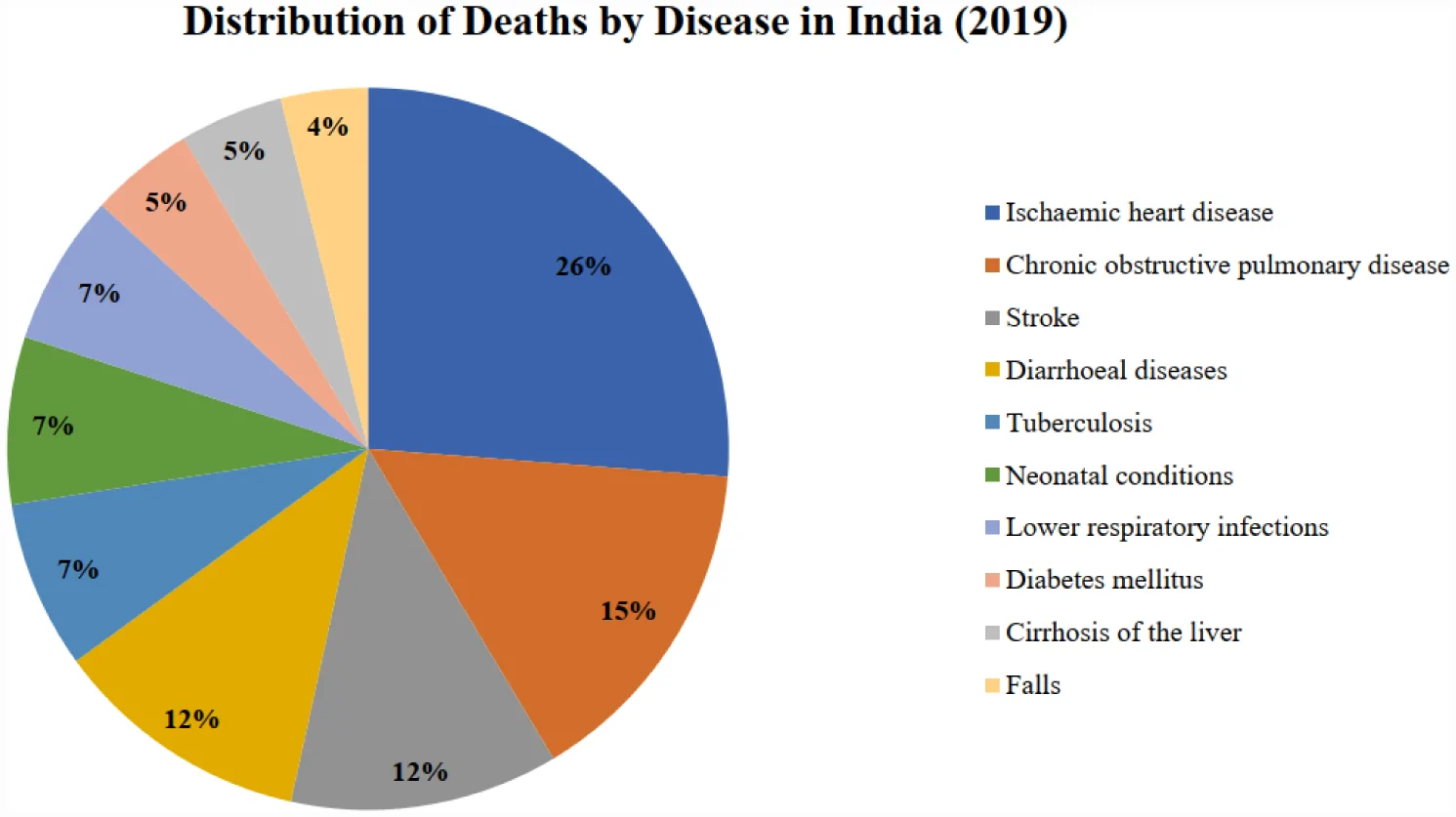
Percentage distribution of deaths by disease in India, 2019 (source: Prepared by authors).

Figure 2 shows that in 2000, the biggest cause of death in India was diarrhea, with 116.33 deaths per 100,000 population annually. Following that, neonatal conditions and ischemic heart diseases were the second and third largest causes of death, causing 97.03 and 84.13 deaths per 100,000 population each year, respectively. Rapid urbanization and industrialization have changed the situation with the change in economic structure. By 2019, India's top three causes of death were all related to air pollution. Ischemic heart disease has become the biggest cause of death, responsible for 110.95 deaths per 100,000 population each year. Chronic obstructive pulmonary disease and stroke were the second and third biggest causes of death, respectively. This shift indicates the significant impact of air pollution on public health in India. The causes of deaths in India for the periods 2000 and 2019 are given in Figures 3 and 4.

**Figure 5.**
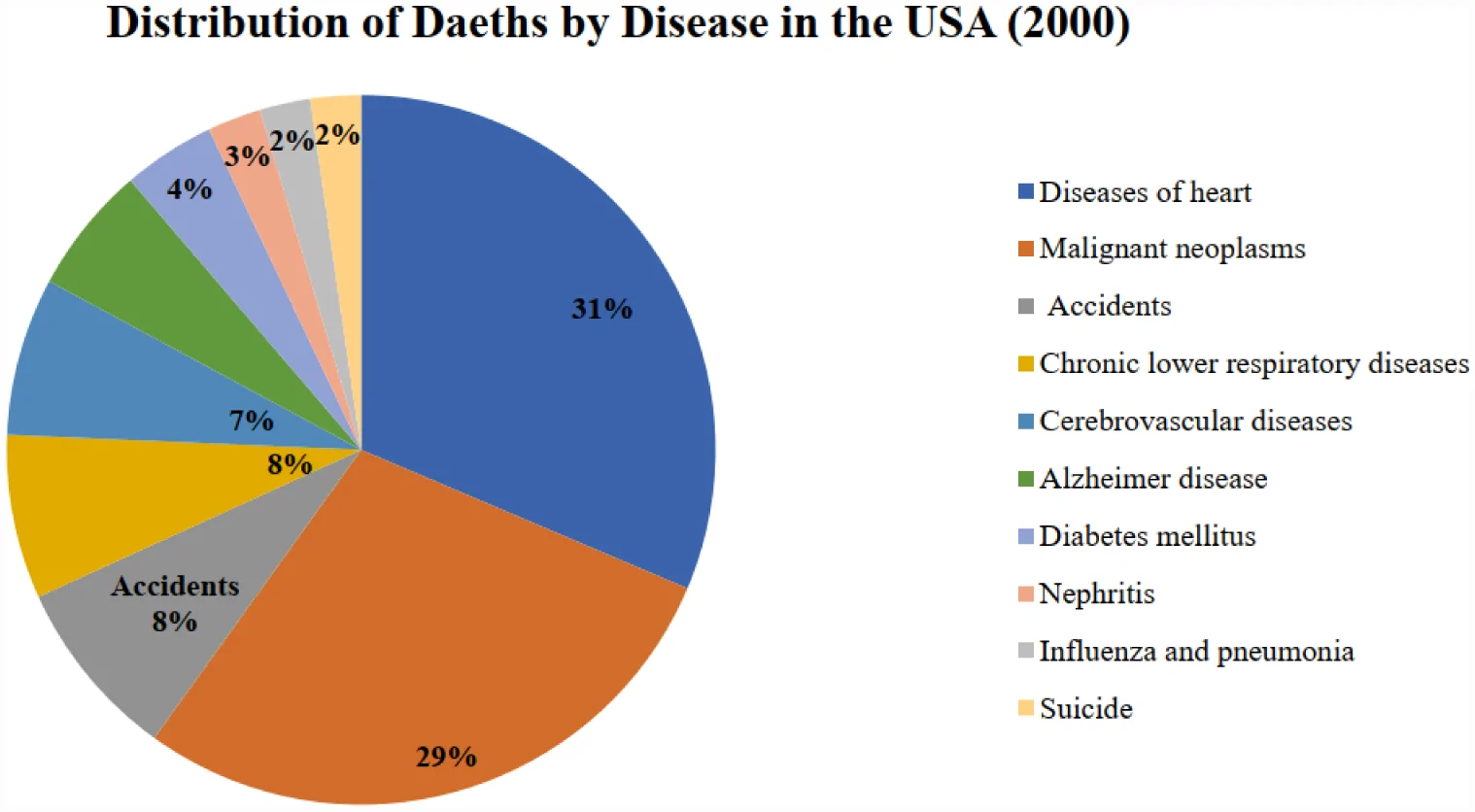
Percentage distribution of deaths by disease in the USA, 2000 (source: Prepared by authors).

**Figure 6.**
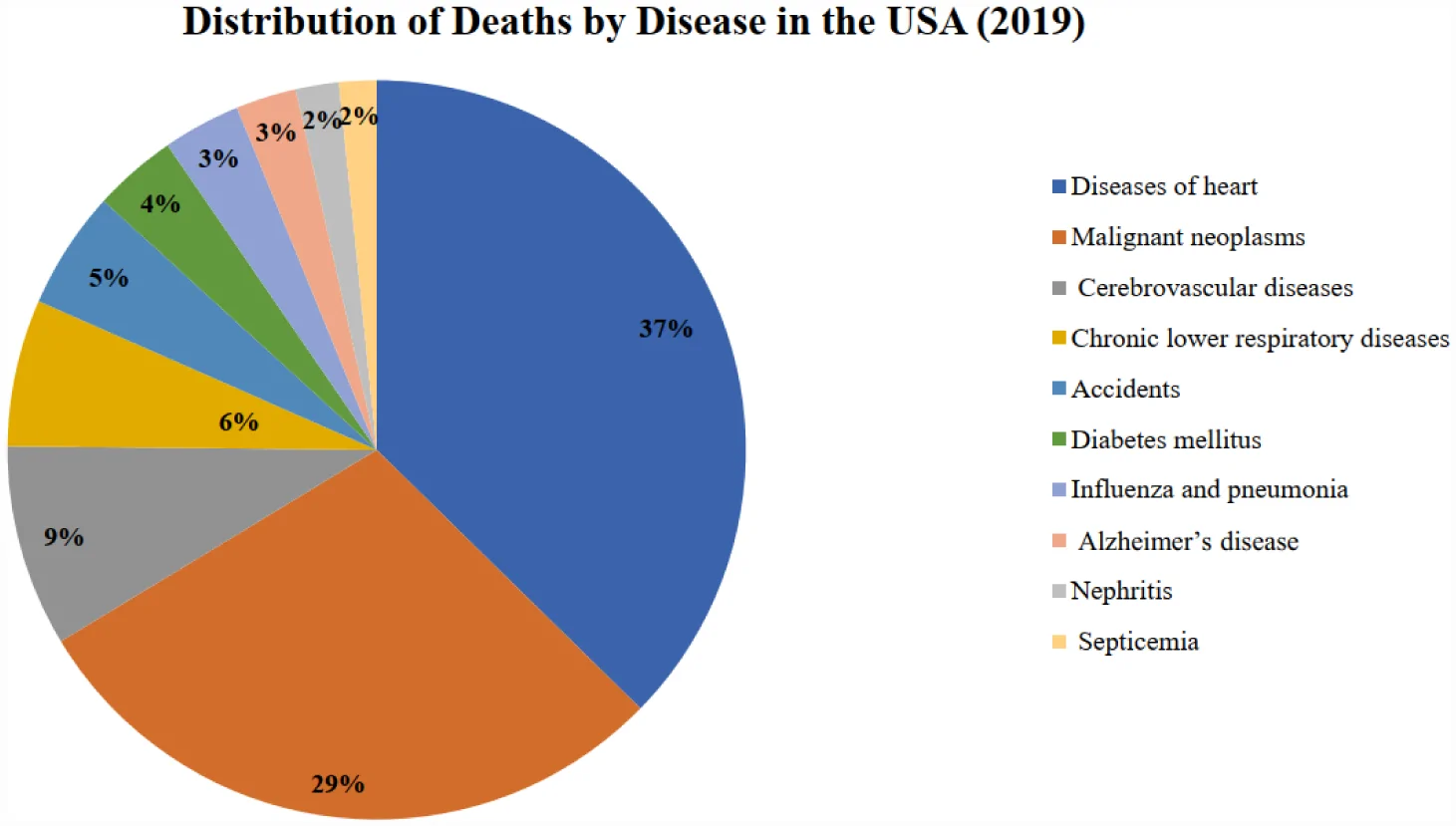
Percentage distribution of deaths by disease in the USA, 2019 (source: Prepared by authors).

From 2000 to 2019, ischemic heart disease was the leading cause of mortality in the USA. Although the USA has not experienced significant alterations in its economic structure, the incidence of premature deaths attributable to ischemic heart disease increased in 2019 compared to 2000. Figures 5 and 6 below provide the causes of death in the USA for 2000 and 2019 for more clarity.

## 4. Discussion

The landscape of air pollution is undergoing a notable transformation. On a global scale, there has been a marked reduction in household air pollution since 1990, owing to the widespread adoption of liquefied petroleum gas and renewable energy sources, which have supplanted traditional biomass fuels like wood, straw, and dung for household heating and cooking purposes [32]. Nevertheless, these advancements are counterbalanced by the surge in ambient air pollution, propelled by the rapid urbanization of megacities, the globalization of industrial activities, the proliferation of pesticides and hazardous chemicals, and the escalating reliance on motor vehicles. The patterns of deaths related to air pollution vary among countries, depending on the level of development. The highest rates of deaths have been seen among countries with low socio-economic development. For instance, in 2019, of total deaths due to air pollution, 89% occurred in low-income countries, showing the devastating situation.

This indicates the large difference between the number of fatalities due to air pollution-related diseases. In developing countries, the people are more exposed to health issues like heart disease, lung cancer, asthma, and stroke compared to the people in developed countries. This exposure can be due to several reasons, such as better air quality, better health facilities and infrastructure, and stricter implementation of environmental laws and regulations that help to improve environmental quality. There can be several reasons for disparity, such as, the level of industrialization and economic growth, political will, bureaucratic efficiency, corruption problems, technological improvements, and infrastructure development [33].

These variables play a vital role in creating the capacity of these nations to reduce pollution and enhance the environment's quality. The well-resourced environmental protection agency and robust public backing enable developed nations are capable to effectively enforce environmental laws and regulations [34]. However, implementing environmental legislation is difficult in developing nations like India due to a lack of funding, ineffective bureaucracy, and conflicting priorities like economic development [35]. To guarantee that environmental laws are implemented and enforced effectively, nations like India must solve these issues and give environmental protection first priority. These elements have the power to help or hurt how well environmental regulations control pollution and safeguard the environment. Furthermore, it is important to take into account the function of regulatory bodies and political institutions in controlling pollution and safeguarding the environment [36].

Additionally, the greater struggle with air pollution in developing countries like India stems from challenges that developed nations have addressed. Developing nations often face tough decisions regarding the balance between economic growth and controlling pollutant emissions. They are less inclined to invest in cleaner energy sources due to the higher cost of renewable resources compared to readily available fossil fuels like coal, which provide cheap polluted energy, crucial for rapid expansion of infrastructure. The rapid expansion of infrastructure in developing nations often hinders the practical installation of cleaner energy alternatives. In contrast, developed nations, having already met most of their basic needs, are more willing to allocate resources to cleaner energy options. For instance, since August 2022, there has been a significant surge in domestic utility-scale clean energy investments, totalling $464 billion, surpassing the cumulative investment in US clean power projects commissioned between 1981 and 2022 [37]. This investment has led to the announcement of 128 new utility-scale clean energy manufacturing facilities, expected to create 43,150 new jobs in the United States and deliver $4.5 billion in savings for over 24 million utility customers.

Comparatively, India invested $4.7 billion in clean energy projects, spread across seventeen 17 initiatives [38]. Despite India's policy allowing 100% foreign direct investment (FDI) in the non-conventional energy sector without prior approval, from 2000 to 2023, the country received $16.26 billion in FDI, which constitutes 2.46% of India's total FDI during this period [39]. However, along with the USA, India is also attempting to control air pollution through clean energy investment. For instance, the clean energy investment in the USA resulted in a reduction of 900 million metric tons of CO_2_ emissions and 1 million metric tons of SO_2_ and NO_x_ from 2019 to 2022, which was equivalent to $249 billions of climate and health benefits [39]. Similarly, India also saved 440 million metric tons of CO_2_ from 2015 to 2020 through clean energy initiatives [40]. Globally, the emissions reduction can save 4.5 million lives over the next fifty years if they are reduced according to the Paris Agreement [41].

## 5. Limitations of the study

In this study, only ozone and PM_2.5_ are considered in ambient air pollution because data for other ambient pollutants are missing. The overall economic loss is also underestimated because we focus only on mortality, and the economic cost of morbidity is not considered. Moreover, we rely on secondary data only, and in India, a large portion of deaths is not reported, so an actual number of deaths can be greater than the data available.

## 6. Conclusions and policy recommendations

This study highlights the vast gap between India and the USA in terms of total premature deaths and economic loss due to air pollution. As per the results, both countries are suffering large economic losses due to premature deaths attributed to air pollution, $34.85 billion in India and $24.76 billion in the case of the USA. However, the per capita loss amounted to $20868 for India and $247,600 for the USA, highlighting the stark disparity in the per capita income. These enormous losses indicate that the governments and people need to take action to reduce air pollution and accomplish their net-zero emissions target. For instance, the Indian government should encourage the public private partnership to increase investment in clean energy sector and promote research and development (R&D) to invent better technologies without cutting the welfare expenditure.

The R&D will help reduce existing infrastructure emissions and build new green infrastructure, like zero house emissions in Japan. Additionally, the existing infrastructure can be repurposed to provide flexibility in support of the rise of solar and wind, while others can be retrofitted either with carbon capture or co-firing with biomass or hydrogen-based fuels. Along with reusing the existing infrastructure, it is also essential to build a new infrastructure as per international environmental standards. For example, the USA uses a system called “ENVISION”, which rates the sustainability and environmental friendliness of new infrastructure.

Additionally, international support is also essential for a country like India, where the investment in clean energy sector from the international sources is relatively muted. India needs to improve investment conditions by reducing the interference of bureaucracy, which takes a long time to be approved. Furthermore, India introduced the Production Linked Incentive (PLI) scheme for clean energy, specifically targeting high-efficiency solar PV modules, aiming to boost domestic manufacturing and offering incentives to manufacturers for producing solar PV modules with high efficiency. This initiative is part of a broader effort to promote renewable energy and reduce the nation's carbon footprint. However, India requires more initiatives like PLI that propel investment friendly environments, since international investment is essential in a country like India where fiscal space is limited.

Moreover, international investment is projected to play a significant role in catalyzing investments in emerging technologies like low-carbon hydrogen. These technologies are often invented in developed economies like the USA, where technology and equipment providers are concentrated. Joint ventures are expected to facilitate the development of pioneering projects in emerging markets like India. Other than these, international institutions that are working to improve the environment's quality need to collaborate with local institutions to better implement the policies and monitoring at the ground level. Doing so will make it relatively easy to monitor the situation and provide jobs for local people, which will motivate them to protect the environment and automatically help reduce air pollution. Along with multilateral institutions and governments, the private sector needs to fund environment-friendly projects such as clean energy projects. Investing in clean energy projects is risky, such as a hydroelectricity projects, as they may not be profitable in when there is a low amount of rain, since, in a hydropower plant, electricity is generated by water. In such cases, the government should provide insurance facilities, which will boost investors' confidence.

Along with these investments, it is also essential for people to take care of minor things that emit a large portion of ambient air pollution, such as vehicular emissions, trash burning, and use of wood stoves. To reduce vehicular emissions, people should use public transport or share their rides so that the number of vehicles on the roads can be reduced. These small steps are also very important, along with investment in better infrastructure and clean energy projects to improve air quality. India must also learn from the USA to implement strict laws and regulations to curb emissions. For instance, the Clean Air Act in the USA is vital in reducing emissions and improving air quality in the country. Finally, it is crucial to address future challenges in dealing with air pollution, such as addressing the transboundary nature of air pollution, which requires international cooperation. Further research is needed to understand the complex interactions between pollutants, climate change, and human health, as well as to develop innovative solutions.

## Use of AI tools declaration

The authors declare they have not used Artificial Intelligence (AI) tools in the creation of this article.
